# Ultrasound classification of traumatic distal biceps brachii tendon injuries

**DOI:** 10.1007/s00256-017-2816-1

**Published:** 2017-11-24

**Authors:** Javier de la Fuente, Marc Blasi, Sílvia Martínez, Pablo Barceló, Carlos Cachán, Maribel Miguel, Carles Pedret

**Affiliations:** 1 Orthopedic Department, Clínica Pakea—Mutualia, San Sebastián, Spain; 2 Department of Fundamental Care and Medical-Surgical Nursing, Faculty of Medicine and Health Sciences (Bellvitge Campus), University of Barcelona, Barcelona, Spain; 3 Àrea d’Estructura i Funció del Cos Humà, Facultat de Medicina i Ciències de la Salut, Universitat Internacional de Catalunya, Barcelona, Spain; 4 Radiology Department, Hospital Universitario de Burgos, Burgos, Spain; 5 Bridgestone Medical Services Hispania, Bilbao, Spain; 6 Ultrasonography Department, Clínica Ercilla—Mutualia, Bilbao, Spain; 7 Human and Embryology Anatomy Unit, Experimental Pathology and Therapeutic Department, Faculty of Medicine and Health Sciences (Bellvitge Campus), University of Barcelona, Barcelona, Spain; 8 Sports Medicine and Imaging Department, Clinica Mapfre de Medicina del Tenis, C/ Muntaner 40, 08011 Barcelona, Spain; 9 Ultrasonography Department, Clinica Diagonal, Esplugues de Llobregat, Spain

**Keywords:** Distal biceps tendon, Ultrasonography, Injury classification, Tendon retraction, External bicipital aponeurosis, Imaging technique

## Abstract

**Objective:**

The present work is aimed at analysing ultrasound findings in patients with distal biceps brachii tendon (DBBT) injuries to assess the sensitivity of ultrasound in detecting the different forms of injury, and to compare ultrasound results with magnetic resonance imaging (MRI) and surgical results.

**Materials and methods:**

A total of 120 patients with traumatic DBBT injuries examined between 2011 and 2015 were analysed. We compared ultrasound results with MRI results when surgery was not indicated and with MRI and surgical results when surgery was indicated.

**Results:**

For major DBBT injuries (complete tears and high-grade partial tears), the concordance study between exploration methods and surgical results found that ultrasound presented a slight statistically significant advantage over MRI (ultrasound: κ = 0.95—very good—95% CI 0.88 to 1.01, MRI: κ = 0.63—good—95% CI 0.42 to 0.84, kappa difference *p* < 0.01). Minor injuries, in which most tendon fibres remain intact (tendinopathies, elongations and low-grade partial tears), are the most difficult to interpret, as ultrasound and MRI reports disagreed in 12 out of 39 cases and no surgical confirmation could be obtained.

**Conclusions:**

Based on present results and previous MRI classifications, we establish a traumatic DBBT injury ultrasound classification. The sensitivity and ultrasound–surgery correlation results in the diagnosis of major DBBT injuries obtained in the present study support the recommendation that ultrasound can be used as a first-line imaging modality to evaluate DBBT injuries.

## Introduction

Distal biceps brachii tendon (DBBT) injuries occur mainly in men aged 40–60 years. The main injury mechanism is an intense extension force applied to the anterior aspect of the forearm with the elbow in an active flexed position [1–4]. The rate of DBBT injuries has been reported to be as low as 1.2 per 100,000 people each year [1, 5–7]. In recent years, however, a higher rate has been reported owing to improvements in diagnostic techniques and anatomical knowledge [8].

The DBBT is composed of two main functionally independent components: a tendon mainly arising from the short head of the biceps brachii muscle and another arising from the long head of the biceps brachii muscle [6]. Both components are surrounded by a single paratenon [9], which can contain peritendinous fluid or effusion surrounding the whole tendon in acute traumatic injuries such as partial tears [10]. The latter must be differentiated from bicipitoradial bursal effusion, found between DBBT and the radial tuberosity, with occasional communications to the interosseous bursae [11]. Besides the DBBT, the biceps brachii muscle also attaches distally by means of the bicipital aponeurosis, also known as the *lacertus fibrosus*, which is a complex fibrous structure that arises medial to the DBBT at the myotendinous junction of the biceps brachii muscle and it runs medial to merge with the antebrachial fascia [5]. It is important to take into consideration this structure when attempting to understand the different clinical behaviour of complete DBBT tears [5]. In the present study, we refer to this aponeurosis as the external bicipital aponeurosis (EBA) to differentiate it from the internal or intramuscular bicipital aponeurosis (IBA).

Complete DBBT tears are most often associated with tendon detachment from the bicipital tuberosity [12]. However, the extent of DBBT injuries and their clinical behaviour varies, ranging from partial to complete tears, which can make diagnosis more complicated and influence therapeutic decision-making [10]. When major injuries are diagnosed, early surgical repair of complete DBBT tears is the recommended treatment to avoid a substantial loss of elbow flexion and supination force [8]. Early diagnosis is thus fundamental for surgical intervention and to facilitate the surgical procedure [8]. Magnetic resonance imaging (MRI) is considered the gold standard in the diagnosis of DBBT injuries. However, this technique presents several limitations, including availability, high average patient waiting times and high costs, which can delay both the diagnosis and treatment of DBBT injuries [8]. Moreover, although it is considered the gold standard, MRI does not always achieve good surgical correlation in cases of partial DBBT tears [10].

The use of ultrasound as an imaging test for diagnosing DBBT injuries is becoming increasingly common. In the past, it had been considered to have less diagnostic value than MRI, mainly because of its lack of accuracy in assessing injuries at the attachment site [13]. However, there is increasing evidence [2, 10] that ultrasound presents many advantages over MRI, e.g. lower cost, greater accessibility, quick contralateral comparison, and the possibility of performing dynamic examinations.

The aim of the present study is to analyse traumatic DBBT injuries diagnosed between 2011 and 2015 in two clinics specialising in occupational medicine. All patients were examined using a standardised ultrasound scanning technique and the ultrasound results were then compared with MRI results and/or with direct-vision results when surgery was indicated. An ultrasound classification system for DBBT traumatic injuries is proposed based on present ultrasound/MRI findings and surgical confirmation.

## Materials and methods

To conduct this study, we selected patients attending a consultation because of an acute traumatic episode with pain in the anterior elbow and in whom morphological and/or sonographic changes in the DBBT were detected by ultrasound. Patient recruitment was performed in two clinics specialising in occupational medicine (Mutualia San Sebastián and Mutualia Bilbao, Spain), between 2011 and 2015. Informed consent was obtained from all patients, in which they authorised the use of the data obtained during their clinical management for the present study performed by researchers from our clinics. The direction board of our institution approved the present study.

All 120 patients underwent a proper anamnesis and clinical examination, which was centred on identifying the injury mechanism, assessment of pain at the antecubital region, assessment of elbow weakness (active and counter-resisted flexion and supination) and palpation of the anterior brachial region to check for a bunching up of the muscle and a palpable defect. Clinical diagnosis is usually easily done in complete and retracted tears, with a bunching up of the muscle and a palpable defect of the DBBT. In partial tears, the clinical diagnosis is more difficult, and the patient may only report pain at the antecubital region and weakness during elbow flexion.

Afterwards, all patients underwent a standardised ultrasound scanning examination (described later in this section) that was designed by two ultrasonographers with more than 20 years’ experience. Ultrasound examinations were performed using an Esaote digital high-resolution machine, model number MyLab70 XVG, and a multi-frequency linear transducer (7–14 MHz). In 81 patients, surgery was indicated and was performed in the same clinic where they were diagnosed. In 43 out of the 81 surgical cases, an additional MRI study was performed. Intraoperative findings made it possible to confirm the ultrasound and MRI results. Additionally, in the 39 patients for whom surgery was not indicated, an additional MRI study was performed to compare and confirm the ultrasound results.

Magnetic resonance imaging studies were performed using a 1.5-Tesla MR Imaging System (MAGNETOM ESSENZA, Siemens Medical Solutions) with a maximum gradient strength of 30 mT/m, a slew rate of 100 mT/m/ms and eight receiver channels. Patients were examined in a prone position, with the upper extremity to be studied extended over the head (also known as the “Superman” position). If the patient was not able to maintain this position, examination was performed in a supine position with the arm resting along the body. A flex coil was positioned wrapped around the elbow. Axial, sagittal and coronal planes were planned orthogonal to the elbow joint after a three-plane localiser. Standard protocol included coronal and sagittal T1 and proton density (PD) with fat saturation (FS) and axial PD and T2-weighted TSE and PD with fat saturation. Sagittal and coronal images were acquired with a field of view (FOV) of 20 × 16 cm and a phase direction proximal to distal. Axial images had a FOV of 17 × 13 cm with a phase direction anterior to posterior.

Standard parameters are indicated for each sequence of the protocol. Some variations were introduced for better imaging of selected cases (e.g. voluminous elbows, patients who moved during examination). Axial PD-T2: TR 2,920 ms, TR 32–96 ms (PD-T2), slice thickness 3 mm, acquisition matrix 256 × 166. Axial PD-FS: TR 2,510 ms, TE 33 ms, slice thickness 3.5 mm, acquisition matrix 320 × 200. Coronal T1: TR 630 ms, TE 11 ms, slice thickness 3.5 mm, acquisition matrix 320 × 240. Coronal PD-FS: TR 2,960 ms, TE 45 ms, slice thickness 3.5 mm, acquisition matrix 320 × 192. Sagittal PD-FS: TR 2,020 ms, TE 36 ms, slice thickness 3.5 mm, acquisition matrix 320 × 200. Sagittal T1: TR 438 ms, TE 13 ms, slice thickness 3.5 mm, acquisition matrix 320 × 240.

Ultrasound, MRI and surgical data were obtained from the reports written by the correspondent specialists. Ultrasound, MRI and surgical images were also reviewed and selected to be further displayed in the present article.

Procedures including anamnesis, clinical, ultrasound and MRI examinations were performed within the first 2 weeks after the traumatic event. Surgery, when indicated, was carried out within the week following the radiological diagnosis.

### Ultrasound scanning technique

The following standardised ultrasound scanning technique was used to examine all patients.

#### Short-axis anterior approach

**Fig. 1:**
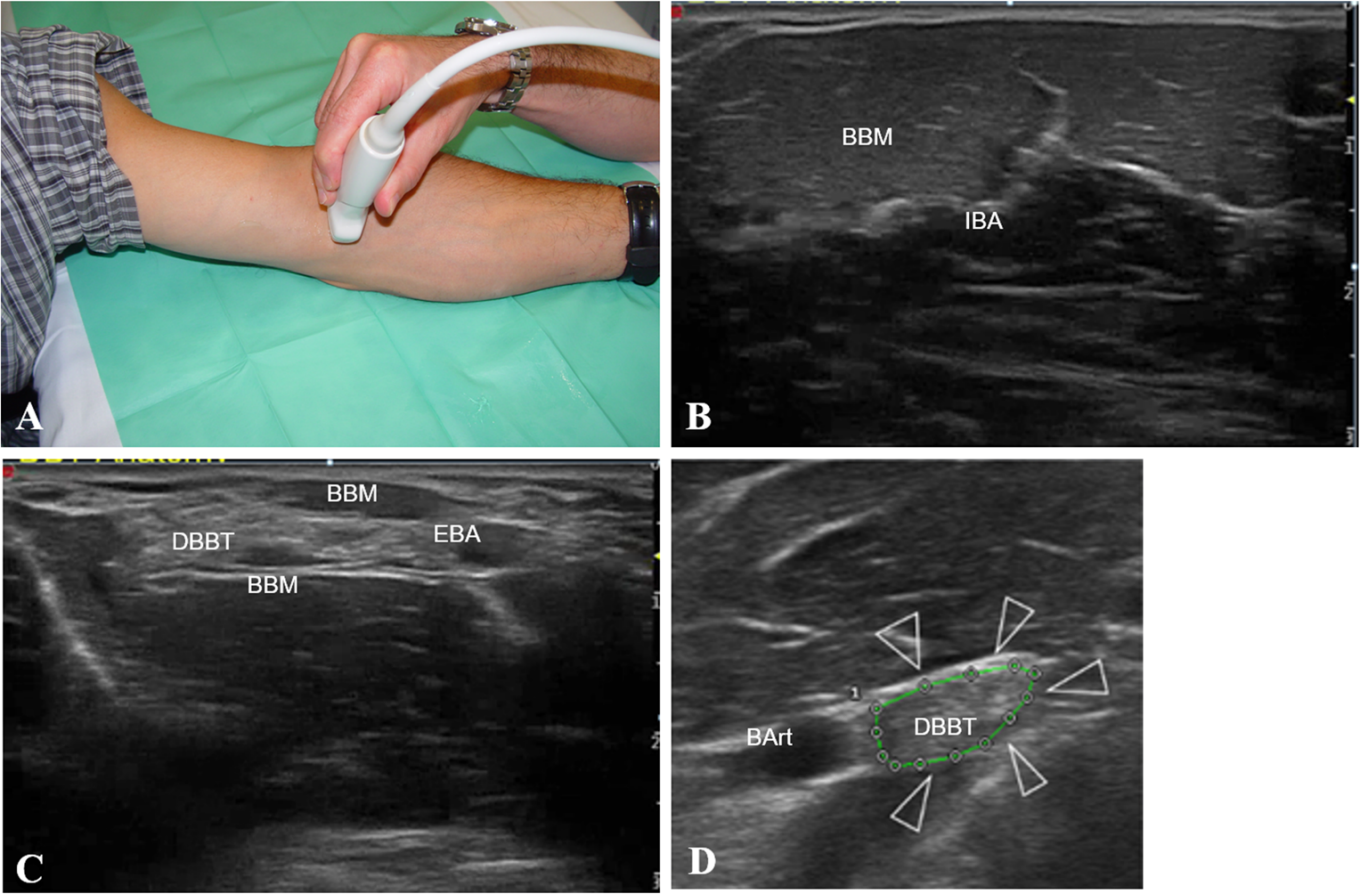
Short-axis anterior approach. **a** The patient is sitting on a stool in front of the examination table resting the elbow in full extension and forearm in full supination. The ultrasound transducer is placed in the axial plane on the anterior half of the arm and then shifted distally. **b** Intramuscular region: ultrasound cross-section view of the internal bicipital aponeurosis (*IBA*) centred within the biceps brachii muscle (*BBM*) belly. **c** Transition region: the distal biceps brachii tendon (*DBBT*) has transitioned to an oval shape, the external bicipital aponeurosis (*EBA*) is observed extending medially with some muscle fibres remaining. It corresponds to the terminal part of the myotendinous junction. **d** Free-tendon: the oval-shaped DBBT has transitioned to a deeper plane towards the radial tuberosity (not visible on the image), and is found lateral to the brachial artery (*BArt*)

The short-axis anterior approach has the elbow in full extension and the forearm in full supination. The ultrasound transducer is placed in the axial plane on the anterior half of the arm. It is then shifted distally as far as the proximal third of the forearm. Three regions corresponding to the normal anatomy of the DBBT should be considered, from proximal to distal: intramuscular (IBA; confluence of the biceps brachii muscle long and short heads), transition (EBA medially and DBBT laterally; myotendinous junction), free-tendon (DBBT; until the radial tuberosity; Fig. 1).

It is important to gradually tilt the probe when examining the free-tendon region, as the tendon becomes deeper, to avoid the anisotropy artefact or to use it to differentiate the tendon from the surrounding soft tissue.

#### Long-axis oblique ulnar approach

**Fig. 2:**
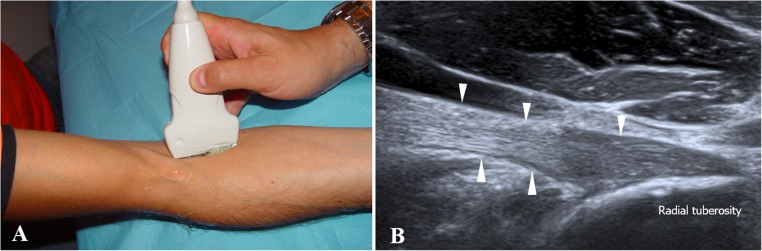
Long-axis oblique ulnar approach. **a** The patient is sitting on a stool in front of the examination table with the shoulder in external rotation, elbow in slight flexion and forearm in full supination. The transducer is placed on the midline of the elbow, at about 20° of ulnar rotation in relation to the upper limb long axis and tilted about 30° medially. **b** Ultrasound image obtained using the approach described in **a**. The fibrillary echotexture of the DBBT (*white arrowheads*) at the attachment site to the radial tuberosity is observed

The long-axis oblique ulnar approach has the shoulder in external rotation, elbow in slight flexion and forearm in full supination. The ultrasound transducer is placed on the midline of the elbow, with an ulnar rotation of about 20 degrees in relation to the upper limb long axis, to locate the brachial artery longitudinally. It is then tilted 30 degrees medially until the DBBT is located (Fig. 2).

It is important to ensure that the transducer is positioned parallel to the DBBT, thus making it possible to analyse the fibrillary echotexture of the tendon even at the attachment site.

### Ultrasound dynamic evaluation

Pronation–supination and elbow flexion–extension dynamic manoeuvres were carried out under ultrasound vision after both the axial and the longitudinal static evaluations.

### DBBT injury ultrasound findings

During ultrasound examinations the following findings were considered and registered, when identified in both the axial and longitudinal views:

1. DBBT sonographic morphological alterations; thickening, thinning, discontinuity and morphometry; diameter, alterations along the whole tendon when observed in both the axial and longitudinal views.
2. DBBT sonographic structural alterations; hyperechogenicity, hypoechogenicity and intratendinous defects along the whole tendon when observed in both the axial and longitudinal views.
3. Liquid effusion around the DBBT when observed in both the axial and longitudinal views.
4. Refraction artefact deep to the tendinous stump when observed in both the axial and longitudinal views, thus indicating complete tears.
5. Absence or hypertrophy of the EBA proximally in the short-axis view.
6. Fibre stretch and tendon movement or absence of the previous during dynamic manoeuvres.

### DBBT injury MRI findings

During ultrasound examinations the following MRI findings (studied in previous literature [14]) were considered and registered:

1. Tendon discontinuity
2. Increased intratendinous signal intensity
3. Peritendinous or intrasheath fluid signal
4. Increased signal intensity: biceps muscle
5. Increased signal intensity in surrounding soft tissue
6. Oedema in radial tuberosity

### Current classification of traumatic DBBT injuries

As a starting point, we used previous MRI-based evidence [14, 15] in terms of DBBT injury classification to allow the different injuries, identified by both MRI and ultrasound, to be divided into the following types:

1. Major injuries: those that require surgical management
  - Complete tear: complete loss of DBBT attachment to the radial tuberosity
    i. Bicipital aponeurosis intact: direct visual confirmation of intact EBA or a retraction gap less than or equal to 8 cm
    ii. Bicipital aponeurosis torn: direct visual confirmation of torn EBA or a retraction gap of more than 8 cm
  - High-grade partial tear: tendon disruption affecting more than 50% of the DBBT attachment
2. Minor injuries: those that do not require surgical management
  - Low-grade partial tear: tendon disruption affecting less than or equal to 50% of the DBBT attachment
  - Elongation injuries or tendinopathies: altered tendon pattern without signs of tearing

### Statistical analysis

Statistical analysis was carried out using the EPIDAT statistical package. For each statistical value, the 95% confidence interval (95%CI) was calculated based on a normal distribution. Fisher’s exact test was used to calculate the difference between qualitative variables. The agreement between exploratory techniques and surgical findings was evaluated using the unweighted Cohen’s kappa (κ) test, and the strength of this agreement using Altman’s scale.

## Results

The population included in the present study had a mean age of 50.53 years (26–68 years). There was a clear predominance of injured males (111, 92.5%) over injured females (9, 7.5%). Both sides were similarly injured (52.50% right elbow and 47.50% left elbow) and both the dominant limb and the non-dominant limb were similarly affected (56.67% injuries in the dominant limb, 43.33% injuries in the nondominant limb).

The number and rate of each injury type, as diagnosed by ultrasound and according to the MRI-based classification system (see Materials and methods, current classification of traumatic DBBT injuries), and considering a new ultrasound-based classification system (see Results, proposed ultrasound classification of traumatic DBBT injuries) was obtained from the analysis of 120 cases (Table 1).

**Table 1:** Number of cases and relative frequency rates for each DBBT injury type, as diagnosed by ultrasound and classified using an MRI-based classification and a new established ultrasound classification system. Ultrasound characteristics of each injury type are specified. Surgery confirmed the diagnosis when it was indicated as part of the treatment (types 3A, 3B, 2B, 2C). Non-surgically confirmed types are strictly based on ultrasound findings

MRI classification system	Ultrasound classification system	Ultrasound description	Number of cases	Relative (%)
No tear	Type 1	Thickened, hypoechogenic, loss of the fibrillary pattern, tendon continuity to the attachment retained, no evidence of torn fibres on the static and dynamic examinations	20	16.66
	Type 1a (Fig. 7)	It particularly affects one of the two DBBT components (short head or long head)	4	3.33
	Type 1b (Fig. 8)	It affects the full thickness of both DBBT components (short head and long head)	16	13.33
Partial tear	Type 2	DBBT thinning, irregular contour, anechoic appearance, partial tendon discontinuity, peritendinous effusion	35	29.16
Low grade (≤ 50 tear)	Type 2a (Fig. 9)	Tendon discontinuity at the attachment site, thinning ≤50% of the total thickness	18	15.00
High grade (>50 tear)	Type 2b (Fig. 5)	Tendon discontinuity at the attachment site, >50% of the total thickness	14	11.66
	Type 2c (Fig. 6)	Tendon discontinuity at the attachment site that affects the full thickness of a single DBBT component (short head or long head), which becomes retracted. A refraction artefact at the level of the tendinous stump is observed	3	2.50
Complete tear	Type 3	Proximal hypertrophic tendon stump with a refraction artefact, snake-like pattern on the long-axis view, absence of tendon fibres at the attachment site, peritendinous effusion	65	54.16
Non-retracted (≤ 8 cm)	Type 3a (Fig. 3)	EBA intact or hypertrophied, with minor tendon retraction	10	8.33
Retracted (>8 cm)	Type 3b (Fig. 4)	EBA tear with marked DBBT retraction, no evidence of EBA continuity	55	45.83

### Surgically confirmed cases: major injuries

Out of 120 injured elbows, 81 were surgically treated owing to the severity of the DBBT tear. This made it possible to confirm and compare the previously obtained ultrasound and MRI results (Tables 2 and 3). The agreement between exploratory techniques (ultrasound or MRI) and surgical findings was (κ) = 0.95—very good—95%CI 0.88 to 1.01 for surgery—ultrasound, and (κ) = 0.63—good—95%CI 0.42 to 0.84 for surgery—MRI. The kappa difference between surgery—ultrasound and surgery—MRI was significant (*p* < 0.01).

**Table 2:** Ultrasound diagnosis and surgical confirmation (κ = 0.95—very good—95%CI 0.88 to 1.01)

			Surgical confirmation				
			Major injuries				Minor injuries^a^
			Complete tears EBA torn	Complete tears EBA intact	High-grade partial tears^b^ at attachment site	High-grade partial tear^b^ affecting a single DBBT component	
Ultrasound diagnosis	Major injuries	Complete tears EBA torn	*54*				
		Complete tears EBA intact		*9*			
		High-grade partial tear^b^ at attachment site		1	*13*		
		High-grade partial tear^b^ affecting a single DBBT component				*3*	
	Minor injuries^a^				1		

Correct diagnoses are shown in italics

^a^All minor injuries were partial tears that affected less than 50% or more of the tendon thickness or without clear signs of tearing (elongations) and therefore were not surgically treated

^b^All high-grade partial tears affected 50% or more of the tendon thickness

**Table 3:** Magnetic resonance imaging diagnosis and surgical confirmation (κ = 0.63—good—95%CI 0.42 to 0.84)

			Surgical confirmation				
			Major injuries				Minor injuries^a^
			Complete tears EBA torn	Complete tears EBA intact	High-grade partial tears^b^ at attachment site	High-grade partial tear^b^ affecting a single DBBT component	
MRI diagnosis	Major injuries	Complete tears EBA torn	*26*				
		Complete tears EBA intact		*4*		2	
		High-grade partial tear^b^ at attachment site		1	*6*		
		High-grade partial tear^b^ affecting a single DBBT component				*1*	
	Minor injuries^a^				3		

Correct diagnoses are shown in italics

^a^All minor injuries were partial tears that affected less than 50% or more of the tendon thickness or without clear signs of tearing (elongations) and therefore were not surgically treated

^b^All high-grade partial tears affected 50% or more of the tendon thickness

#### Ultrasound results

**Fig. 3:**
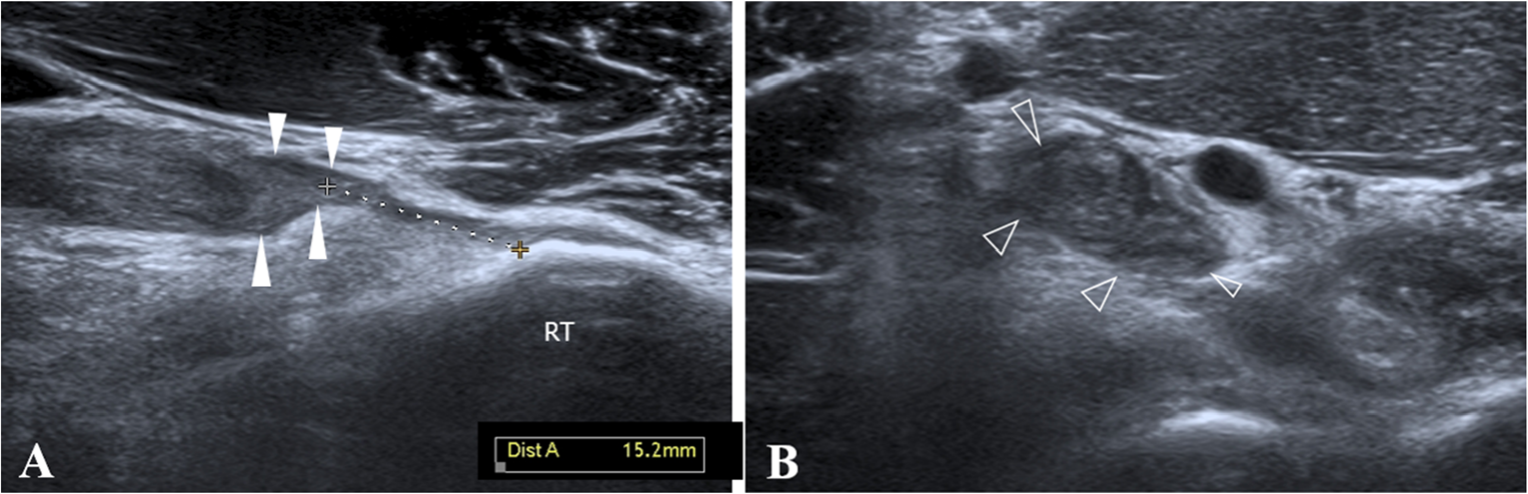
Ultrasound view of a type 3A injury (complete non-retracted tear due to intact EBA). **a** In the long-axis view, a DBBT tear with a mild retraction of the DBBT is observed and confirmed by measuring the gap between the stump (*white arrowheads*) and the radial tuberosity (*RT*). **b** Short-axis view of **a**. Cross-section of the DBBT stump (*arrowheads*) showing tendon hypertrophy and an altered echotexture

**Fig. 4:**
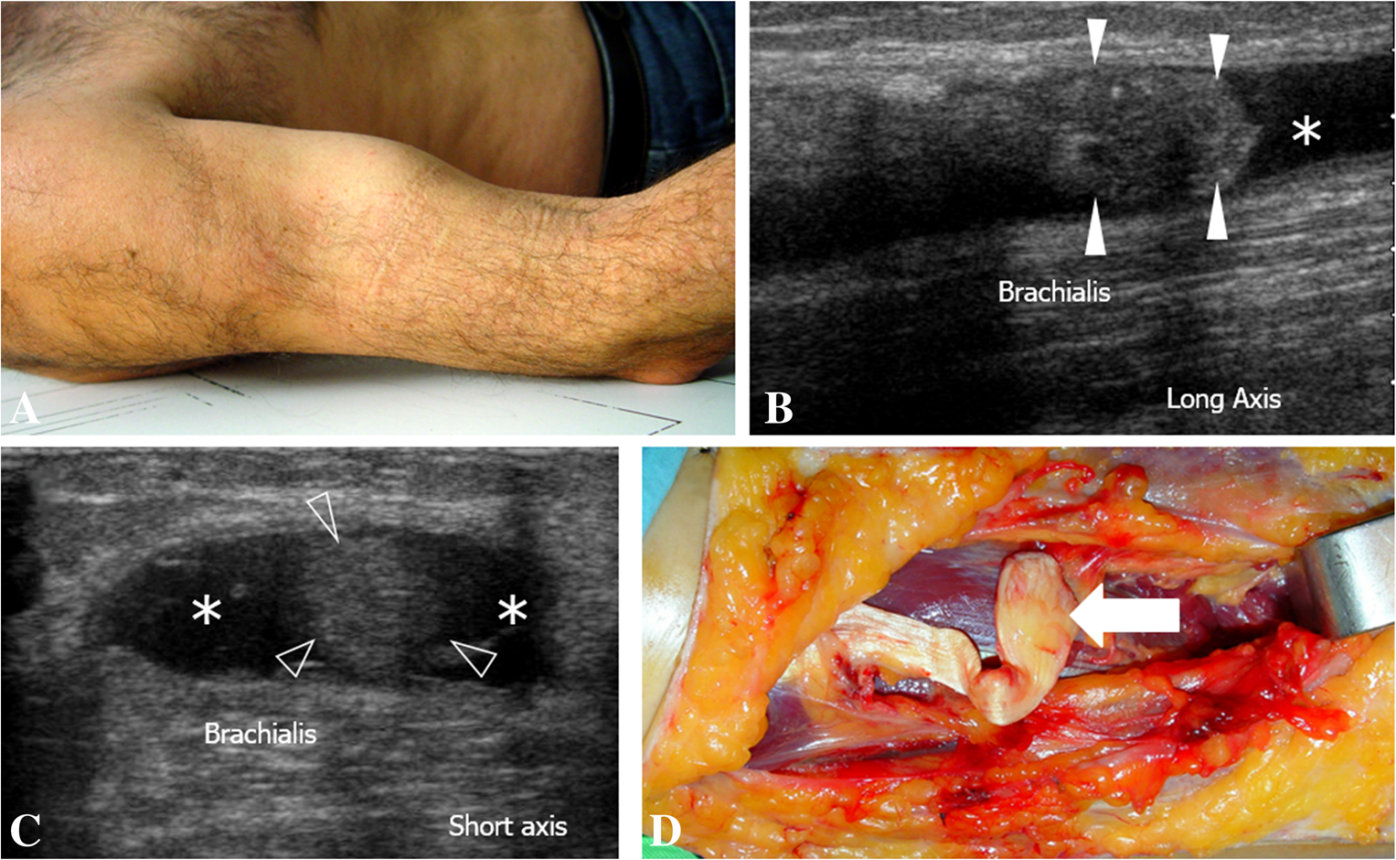
Ultrasound view of a type 3B injury (complete retracted tear due to associated EBA tear) and images of surgical confirmation. **a** During the physical examination, a clear tumefaction corresponding to the retracted DBBT and muscle (also known as the Popeye sign) is observed at the anterior aspect of the arm. **b** In the long-axis view, the DBBT stump (*white arrowheads*) presents a wavy aspect. A haematoma (*asterisk*) is observed distal to the DBBT stump. **c** Short-axis view of **b**. Cross-section of the DBBT (*arrowheads*) surrounded by a haematoma (*asterisk*). **d** Surgical confirmation image of **b** and **c**. The DBBT and major muscle retraction are verified. The wavy pattern of the DBBT stump (*white arrow*) correlates with that of ultrasound in image **b**

In 64 out of 81 surgically treated patients, a complete tear diagnosis was confirmed intraoperatively (Figs. 3 and 4). Sixty-three out of 64 patients had previously had a correct ultrasound diagnosis, whereas the remaining case was misinterpreted as a high-grade partial tear. Ultrasound sensitivity for DBBT complete tears was S = 0.98, 95%CI: 0.91 to 0.99.

**Fig. 5:**
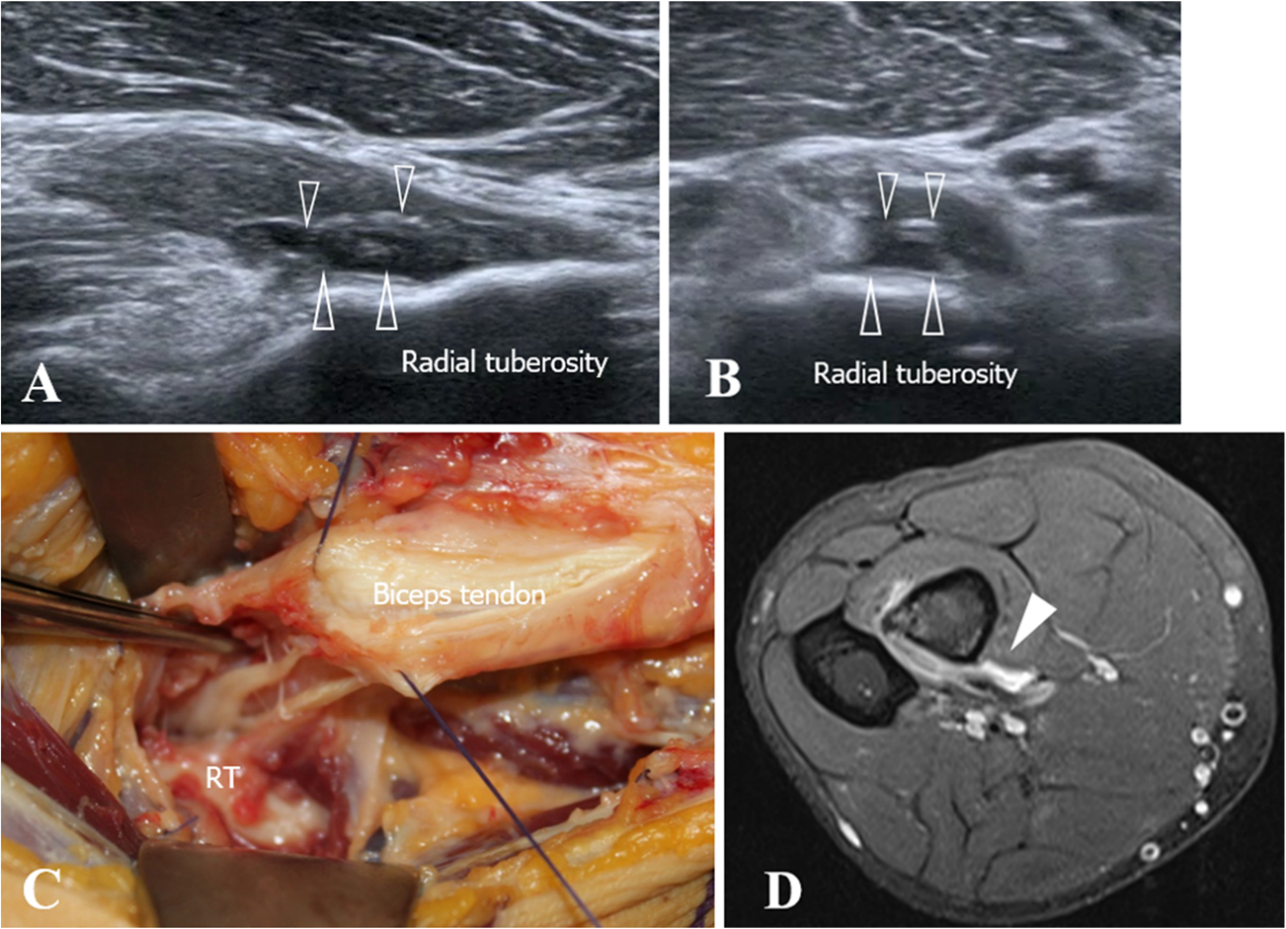
Ultrasound and MRI views of a type 2B injury (high-grade partial tear at the attachment) and image of surgical confirmation. **a** In the long-axis view, a high-grade partial detachment (*arrowheads*) affecting more than 50% of the DBBT (biceps tendon) is observed adjacent to the radial tuberosity. The most superficial *arrowheads* show a hyperechogenic line that corresponds to the torn tendon interface. The defect percentage is calculated by considering tendon thinning, not by considering the amount of anechoic fluid, as its image can be easily altered owing to transducer pressure over the examination region. **b** Short-axis view of **a**. The important anechoic defect demonstrates that most tendon fibres are torn (*arrowheads*). **c** Surgical image correlating with images **a** and **b**. The forceps point to the attachment of some remaining DBBT (biceps tendon) fibres to the radial tuberosity (*RT*). **d** Elbow MRI, axial PD with FS-weighted sequence. An intratendinous hyperintense signal (*arrow*), similar to that of fluid, shows a tendon defect affecting more than 50% of the DBBT and therefore compatible with a high-grade partial detachment type 2B

**Fig. 6:**
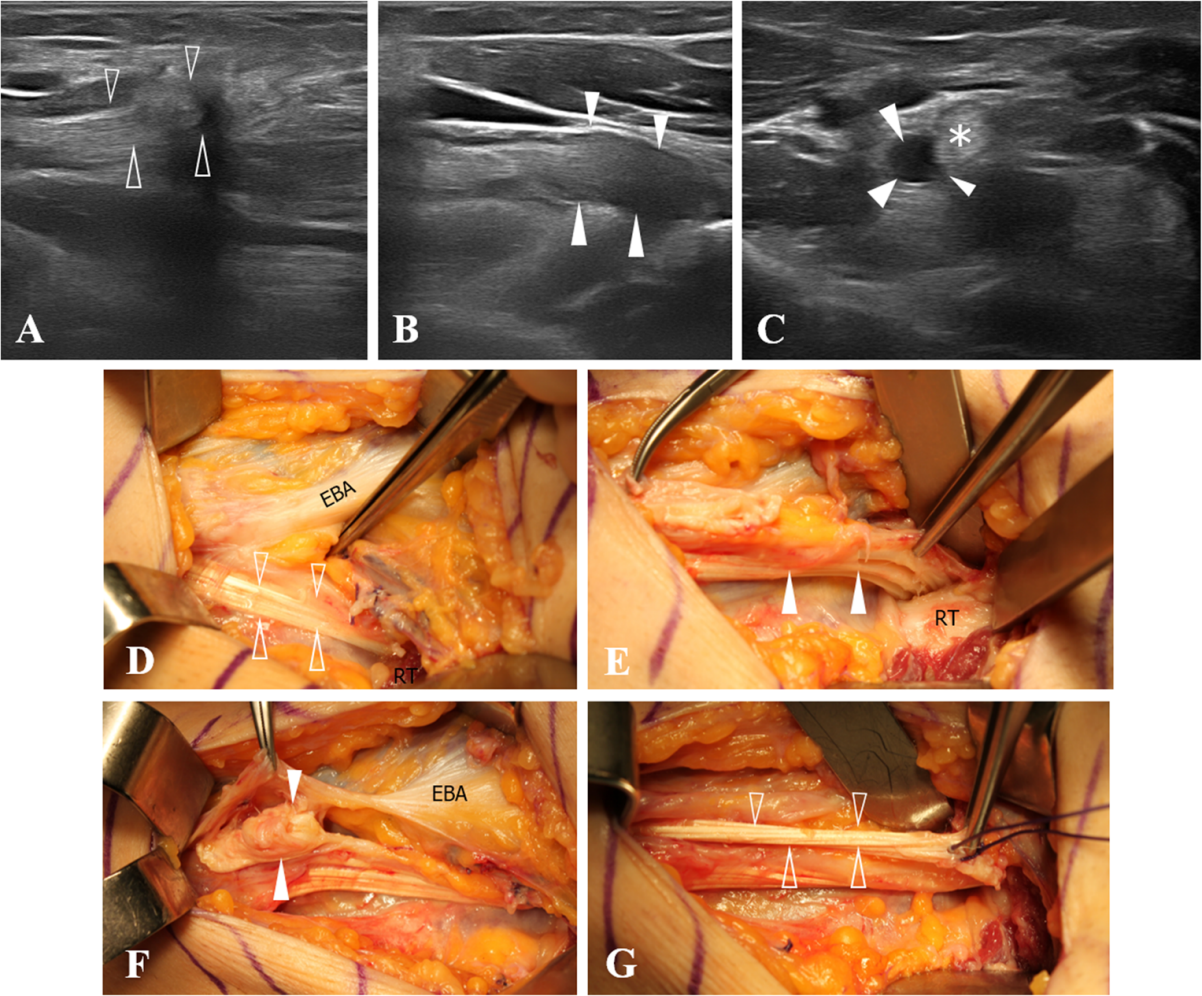
Ultrasound view of a type 2C injury (high-grade partial tear with retraction of one of the DBBT components) and images of surgical confirmation. **a** In the long-axis view, the DBBT signal disruption and a refraction artefact at the level of the tendon stump indicate a tendon tear (*arrowheads*) affecting the short head component of the DBBT, which has detached and retracted from the radial tuberosity. **b** However, when slightly shifting the transducer, it is possible to verify that the other DBBT component, corresponding to the long head, remains intact (*arrowheads*). **c** Short-axis view of **a** and **b**. Two oval-shaped elements can clearly be observed. The anechoic image (*arrowheads*) corresponds to the absent, torn and retracted short-head DBBT component (*arrowheads*), whereas the hyperechogenic image corresponds to the preserved long-head DBBT component (*asterisk*). **d** Some DBBT fibres (*arrowheads*) can be observed, as they are found in their normal path and retain tension. In addition, the external bicipital aponeurosis (*EBA*) can be observed expanding medially to the medial epicondyle muscle mass. **e** When the DBBT fibres identified in **d** (*arrowheads*) are pulled with the forceps, their attachment to the radial tuberosity (*RT*) can be verified. **f** The paratenon has been sectioned at the level of the proximal EBA and the tendon stump corresponding to the torn DBBT short-head component (*arrowheads*) is exposed. **g** The retracted torn DBBT short-head component can be pulled as far as its footprint on the radial tuberosity (*arrowheads*), thereby verifying the complete rupture of this component

In the remaining 17 surgically treated patients the diagnosis of a high-grade partial tear, either at the attachment site or affecting one of the two DBBT components that became retracted, was confirmed intraoperatively (Figs. 5 and 6). Sixteen out of these 17 cases had previously been correctly identified by ultrasound, whereas the remaining case was misinterpreted as a minor injury, most likely an elongation injury without tendon-tearing (Table 2). Ultrasound sensitivity for high-grade partial tears S = 0.94, 95%CI: 0.73 to 0.99.

#### MRI results

Forty-three out of 81 surgically treated patients were additionally examined by MRI. In 31 out of these 43 cases, a complete tear diagnosis was confirmed intraoperatively. Thirty out of 31 cases had previously been correctly identified by MRI, whereas 1 out of 31 was misinterpreted as a high-grade partial tear. MRI sensitivity for complete tears was S = 0.96 (95%CI: 0.84 to 0.99).

In the remaining 12 MRI cases, a high-grade partial tear diagnosis was confirmed intraoperatively (Fig. 5). Seven out of these 12 cases had previously been correctly diagnosed by MRI whereas 5 had been misinterpreted, 3 as minor injuries (tendinopathies) and 2 as complete tears (Table 3). MRI sensitivity for high-grade partial tears was S = 0.58 (95%CI: 0.32 to 0.81).

### Cases without surgery: minor injuries

Out of 120 injured elbows, 39 were conservatively treated owing to a minor DBBT injury. All of these non-surgical cases were examined by both ultrasound and MRI. The cases in which the ultrasound and MRI diagnoses coincided or differed were registered (Table 4).

**Table 4:** Magnetic resonance imaging concurrence or disagreement with ultrasound in the evaluation of non-surgical cases

			MRI diagnosis		
			Major injuries	Minor injuries	
				Minor partial tear at attachment site	Elongation injuries/tendinopathies
Ultrasound diagnosis	Major injuries		*1*		
	Minor injuries	Minor partial tear at attachment site	1	*13*	4
		Elongation injuries/tendinopathies		7	*13*

Correct diagnoses are shown in italics

The case of a complete tear (major injury) in which ultrasound and MRI diagnosis agreed corresponds to a patient who refused to undergo surgery and therefore no surgical confirmation could be obtained

**Fig. 7:**
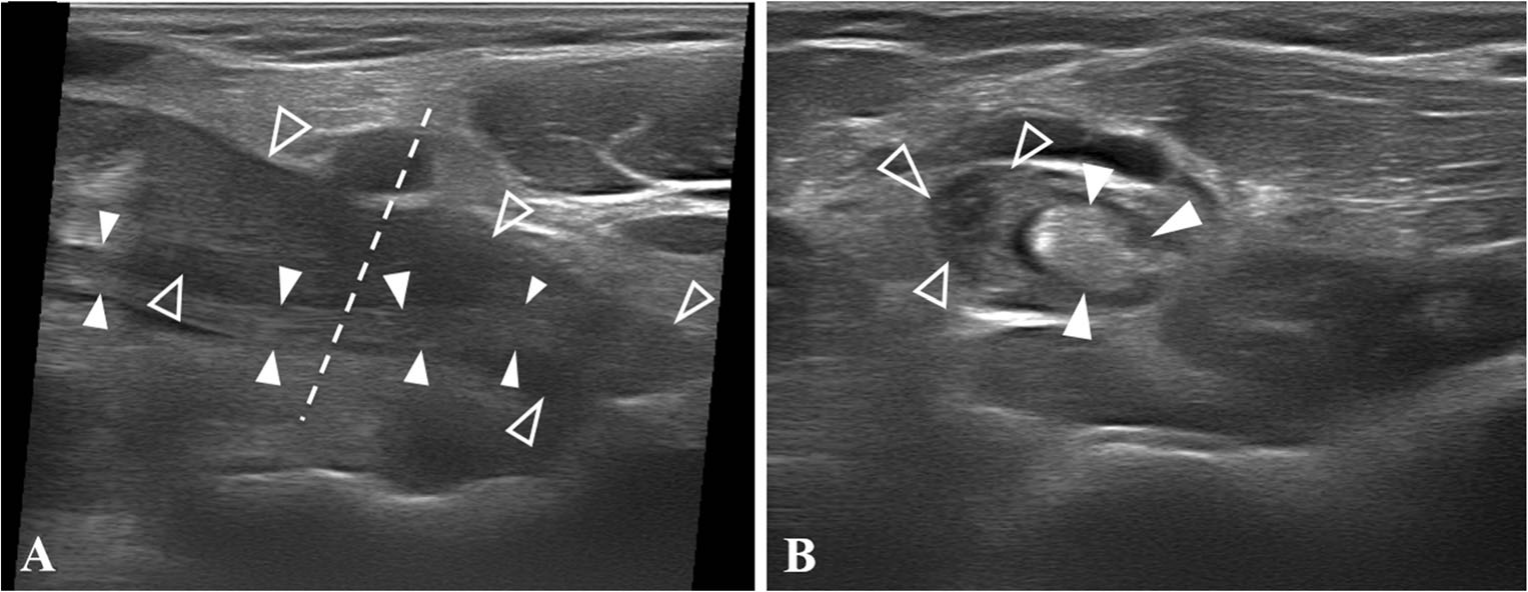
Ultrasound view of a type 1A injury (elongation or traumatic injury without evidence of tendon-tearing affecting mainly one of the two DBBT components). **a** In the long-axis view, different echotextures and morphologies are observed in the elongated long-head component (*empty arrowheads*), i.e. hypertrophic and hypoechogenic, and the normal short-head component (*white arrowheads*), i.e. normal thickness and fibrillary pattern. As the DBBT rotates distally, both components cross over each other on the longitudinal axis. The *dashed line* shows the point where image **b** was obtained. **b** Short-axis view of **a**. The same findings are observed in a cross-section view. An anechoic interface between the two DBBT components is observed. We have considered it a detachment of both components at the level of the endotenon septum owing to shear stress when the long-head component was elongated

**Fig. 8:**
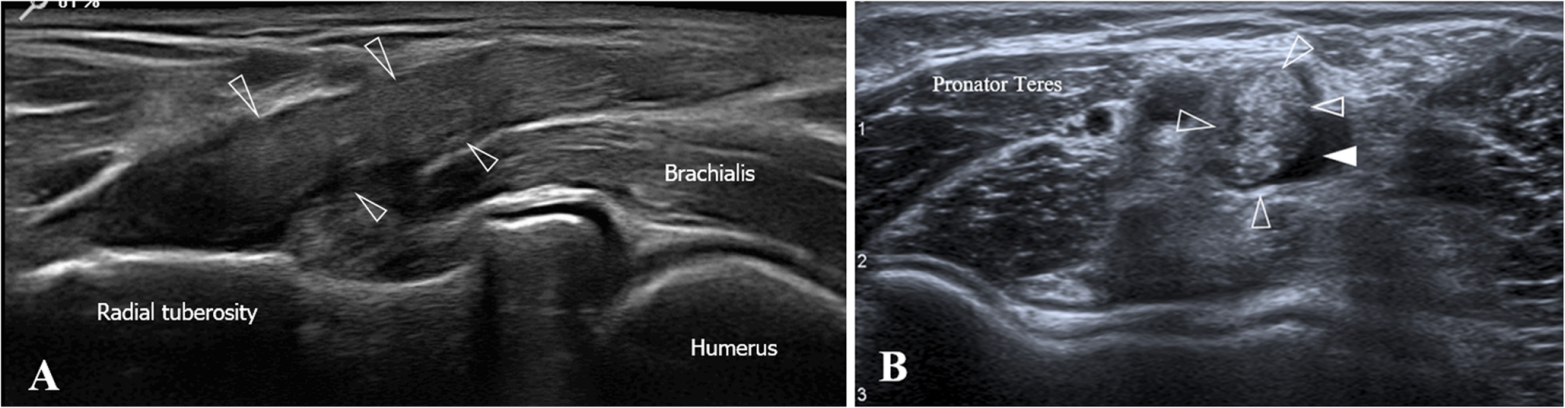
Ultrasound view of a type 1B injury (elongation or traumatic injury without evidence of tendon tearing). **a** In the long-axis view, the DBBT (*empty arrowheads*) is uniformly thickened and hypoechogenic, with a loss of the fibrillary pattern affecting its whole thickness and length. However, there are no signs of tendon tearing. **b** Short-axis view of **a**. The DBBT cross-section shows that the alteration affects both tendon components (*empty arrowheads*), which are observed as being thicker and having an altered echostructural pattern. Also, a localised peritendinous effusion is observed (*white arrowhead*)

**Fig. 9:**
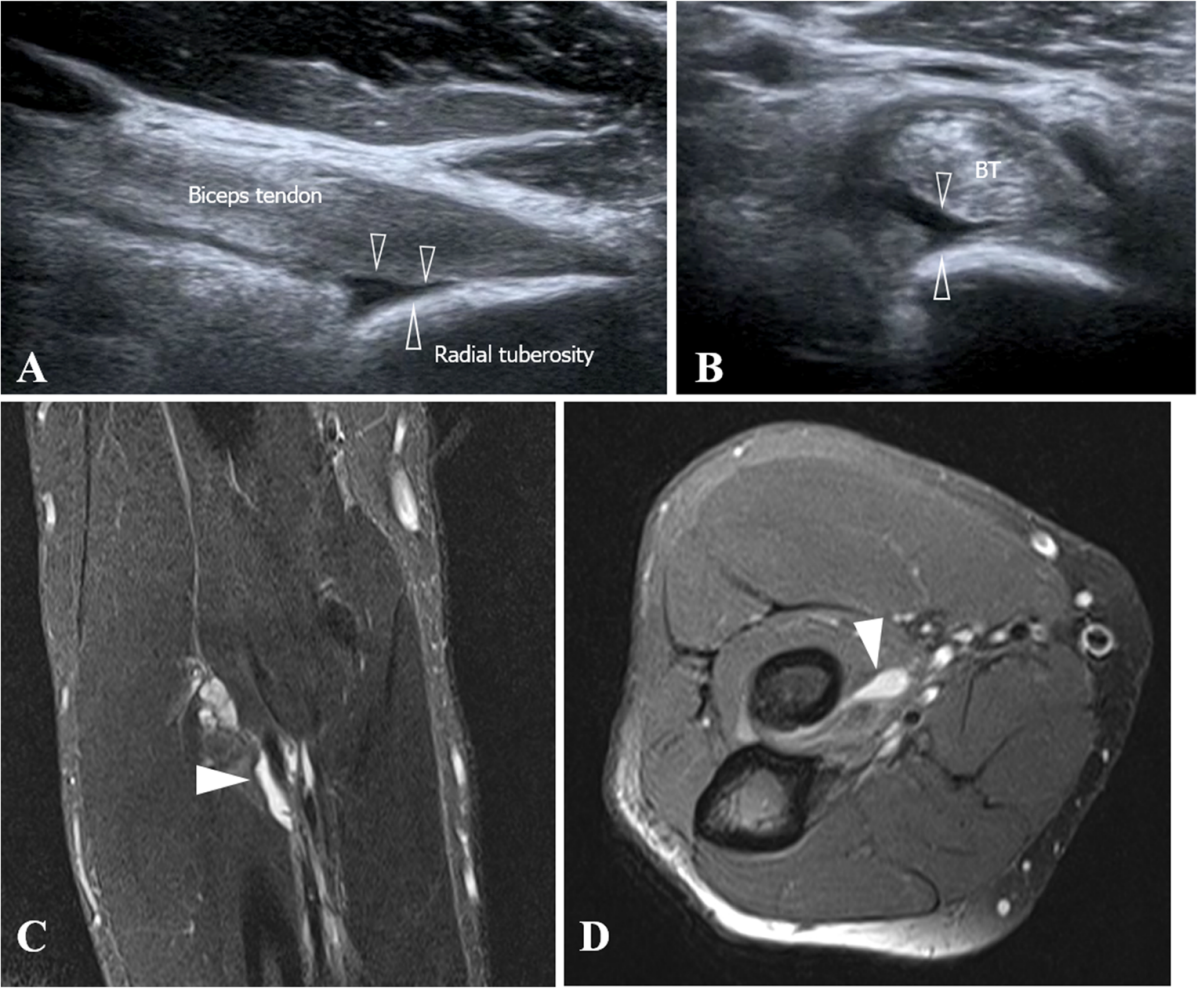
Ultrasound and MRI views of a type 2A injury (low-grade partial tear at the attachment). **a** In the long-axis view, a minor partial detachment (*arrowheads*) affecting less than 50% of the DBBT (biceps tendon), is observed just adjacent to the radial tuberosity. The minor partial detachment can be differentiated from other pathological processes as it is presented as a mild focal thinning of the DBBT just adjacent to the radial tuberosity. The tendon defect is then filled with anechoic fluid. **b** Short-axis view of **a**. The cross-section view also demonstrates the minor partial detachment (*arrowheads*) and a better view of a mild peritendinous effusion that could also be interpreted as a bicipitoradial bursitis secondary to synovial irritation by bleeding after the tear. **c**, **d** Elbow MRI, coronal and axial PD with FS-weighted sequences (respectively). DBBT thickening and contour alteration can be observed at the attachment site. At that point an intratendinous hyperintense signal (*arrow*), similar to that of fluid, shows a tendon defect affecting less than 50% of the DBBT and therefore compatible with a low-grade partial detachment type 2A

The ultrasound and MRI diagnoses coincided in 27 out of 39 cases, 13 of which related to tendon elongation (Figs. 7 and 8), 13 to low-grade partial tears (Fig. 9) and 1 to a complete tear. The latter is a major injury; however, the patient refused surgical treatment and therefore we included it in this section of the results.

The ultrasound and MRI diagnoses differed in 12 out of 39 cases. In 7 out of these 12 cases, ultrasound identified DBBT elongation, whereas MRI identified low-grade partial tears. In 4 out of 12 cases, ultrasound identified a low-grade partial tear, whereas MRI did not identify a tendon tear, but rather an elongation injury. In the remaining case, ultrasound identified a low-grade partial tear, whereas MRI identified a complete tear.

### Proposed ultrasound classification of traumatic DBBT injuries

We propose an ultrasound classification of DBBT traumatic injuries (Table 1) based on the analysis of the ultrasound findings obtained and contrasted in the present study. We have defined a specific group for complete DBBT tears (type 3), with two subgroups, depending on whether the EBA remains intact and/or whether retraction of the DBBT is minor; less than or equal to 8 cm (type 3A; Fig. 3), or the EBA is torn and/or retraction is significantly greater; over 8 cm (type 3B; Fig. 4). Second, we have defined a group for partial tears (type 2) depending on the localisation of the tear and the percentage of fibres torn into low-grade (less than or equal to 50%) partial tears at the attachment site (type 2A; Fig. 9), high-grade (over 50% of tendon fibres torn) partial tears at the attachment site (type 2B; Fig. 5), and high-grade partial tears at the attachment site affecting the whole thickness of either the long-head or the short-head component of the DBBT, which becomes retracted (type 2C; Fig. 6). Finally, although we could not obtain surgical confirmation to fully differentiate low-grade partial tears at the attachment site (type 2A) from elongations or tendinopathies, and MRI disagrees with ultrasound in a number of cases (Table 4), we propose another injury type that involves altered morphological (thickened) and echotextural parameters (wavy pattern), but without evidence of DBBT torn fibres after a trauma, the elongation injury (type 1). Moreover, when analysing this type in the short-axis view, it can be observed how either only the short head or the long head of the DBBT may be affected (type 1A; Fig. 7) or the whole tendon thickness (type 1B; Fig. 8).

## Discussion

According to some authors [6, 16, 17], DBBT injuries account for 3% of all biceps brachii-related injuries, with a higher incidence rate among men aged 40–60 years old. It is associated with a sudden and intense extension force applied to the elbow when it is pre-set in an active flexed and supinated position [1–4]. DBBT avulsion is considered a rare injury, with an annual rate of 1.2 injuries per 100,000 people [4–7]. However, a number of studies conducted in recent decades have reported a clear increase in the DBBT injury rate and a reduction in age at the time of injury [1, 3, 4, 18, 19]. In the present study, 120 cases with a traumatic DBBT injury were diagnosed by clinical criteria and ultrasound examination. These patients were part of a group of 285,000 people with company-paid health insurance, and the annual rate reported was therefore 8.5 injuries per 100,000 people. Although this rate is clearly higher than that previously reported, it should be considered in the context of active working people with a mean age of 50.5 years.

Traumatic DBBT injuries occur almost exclusively in men; in fact, the first description of an injured woman in the literature was reported in 2005 [7]. This major disparity in the incidence rate between the genders is further reported in the recent literature [14] and reflected in our study, as just 9 women were diagnosed DBBT injuries, compared with 111 men. Furthermore, no women required surgical treatment, whereas 81 men did. Therefore, not only are these injuries much less frequent in women, but they also seem to be less severe. In terms of injury side and upper limb dominance, our study contradicts the previous literature, which reports that up to 86% of cases involved injuries to the dominant upper limb [7, 8, 20], as opposed to 56.67% of our cases. Moreover, we report that 52.5% of the injuries affect the right upper limb and 47.5% the left upper limb, with no statistically significant differences.

Distal biceps brachii tendon injuries may present in many different ways depending on injury severity, which may be associated with the intensity of the traumatic event that caused them. In this regard, complete DBBT tears have been associated with a traumatic event with a high counter-resisted force (40 kg or more) to the elbow in a pre-set 90° flexion position [21], whereas associated partial tears have been associated with a trauma of lesser intensity or even with cases with no history of a previous traumatic event [22], suggesting pre-existent tendon degeneration. Although we could not quantify it, all patients in the present study independently of which grade of DBBT injury they had, referred to a high-intensity, sudden, counter-resisted force on the anterior aspect of the elbow while performing different tasks at their workplaces. At the exact moment of the traumatic event on the elbow, patients referred to an acute pain in the anterior aspect of the elbow, a tearing sensation or even an audible “click”.

The most severe DBBT injury is the complete retracted tear (Fig. 4), which in our study accounted for about 45% of the DBBT injuries reported (Table 1). Clinically, they are easily identified because of an obvious deformity (distal arm mass or Popeye sign), ecchymosis and elbow flexion weakness after a traumatic episode [1, 7, 22, 23]. Also, various clinical tests [24, 25] have been described to assess this type of DBBT injury. However, the remaining DBBT injuries, accounting for about 55% in our study (Table 1), such as complete tears without or with minor retraction and partial tears, are only characterised by elbow pain in many cases [14]. Therefore, injury mechanism and clinical diagnosis should always be considered alongside complementary imaging tests, especially in cases without clinical manifestation of a non-typical complete retracted tear.

At the moment, MRI has been postulated to be the gold standard for identifying the different types of DBBT injury [14, 20], but the costs and delays associated with MRI, over 10 days in 50% of cases [26, 27], may have a significant clinical impact on surgical outcomes [12, 26, 27]. Surgical treatment of complete DBBT tears is widely accepted as the treatment of choice [1, 3, 6, 18, 21, 28–31] and when performed shortly after an early diagnosis, it eases surgical reattachment and avoids complications such as muscle retraction, disorganised scar tissue formation, shortening and degeneration [3, 30, 32]. As ultrasound involves lower costs and is more accessible than MRI, an early systematic ultrasound examination of the DBBT could be complementary or an alternative to MRI. However, ultrasound examination of the DBBT is not straightforward, and even Brasseur described it as “diabolical” [33].

The anatomical characteristics and problems associated with ultrasound examination of this tendon have brought about the need for different ultrasound approaches or windows to be proposed in recent years. Most authors recommend a transverse or short-axis view to examine the anterior aspect of the elbow. The only technical difficulty associated with this approach is the anisotropy artefact due to the obliquity of the DBBT as it reaches the deeper planes towards the radial tuberosity; the examiner can easily avoid or exploit this by progressively tilting the transducer [9, 10, 33–35]. The longitudinal or long-axis view is technically much more difficult, and several ultrasound approaches or windows have therefore been suggested. These are the lateral approach [36], the posterior approach with the forearm in prone position [37], the medial approach [38] and the anterior approach [34, 36], which is the most advisable. When dealing with the DBBT, however, it is necessary to bear in mind that a combination of approaches or windows and the fact that it is important to study the whole length of the tendon up to its attachment to the radial tuberosity, as many injuries can occur at the attachment site, is required to make a sound diagnostic decision [10, 34]. We have standardised and systematised an ultrasound technique by combining the two most accepted approaches mentioned above (anterior transverse or short-axis and anterior longitudinal or long-axis), with a slight modification. The modification made was to the long-axis anterior approach and we termed it the anterior ulnar oblique approach. We believe that this modification enhances examination of the distal attachment, as it allows the tendon attachment to be viewed most parallel to the ultrasound transducer, obtaining a better image of the fibrillary tendon echotexture.

Most complete tears of the DBBT occur as tendon avulsions 1–2 cm proximal to the attachment to the radial tuberosity, which corresponds to a hypovascularised portion of the tendon that corresponds to the fibrocartilaginous transition of the enthesis [8, 21, 22, 39]. Complete tears, as identified by MRI, consist of tendon discontinuity with a proximal thickening of the proximal stump and an abnormal signal intensity [5, 13, 14, 25]. Ultrasound short-axis view findings are very similar to those obtained by MRI, consisting of tendon discontinuity, absence of the distal tendon in its normal position, a thickened proximal stump and effusion within the paratenon (Figs. 3 and 4) [14, 20]. In the present series, which consisted of 65 patients with a complete DBBT tear, we correctly identified 64 out of 65 cases using ultrasound (Table 2), which demonstrates a sensitivity for complete retracted tears of over 95% (S = 0.98 95%CI: 0.91 to 0.99). These results are very similar to those obtained by MRI in both the present study (Table 3), in which 31 out of 32 complete tears were correctly identified (S = 0.96 95%CI: 0.84 to 0.99), and in the previous literature [14]. In our experience, they were very easily identified in the short-axis view, using the anterior approach, as in all correctly diagnosed cases, the sudden loss of the typical ovoid-shaped DBBT image when scanning from proximal to distal was very noticeable (Figs. 3 and 4). In this regard, we believe that short-axis scanning is the most effective examination for identifying such injuries. The long-axis view allows the short-axis findings to be corroborated and the degree of DBBT retraction to be measured (Fig. 3), a crucial step in terms of surgical planning. Finally, the dynamic pronation–supination examination confirms this type of injury if movement of the proximal retracted tendon stump is not observed during these manoeuvres.

As mentioned earlier, complete tears may be associated with a torn or an intact EBA. On MRI, retraction of the DBBT has been found to be an indirect sign for assessing this matter, as an 8-cm DBBT retraction has been correlated with an EBA tear [15]. This important gap in complete tears with a torn EBA most likely account for the arm deformity typically observed in association with these injuries that may otherwise be minimised or even absent in complete tears with an intact EBA [13]. Moreover, patients with the latter retain a residual ability to flex the elbow and supinate the forearm [14]. An intact EBA may even have consequences in post-surgical patient management, as it enables a more aggressive approach to be taken in post-surgical rehabilitation [8]. In our series, on both MRI and ultrasound, we succeeded in correctly identifying 10 patients with complete DBBT tears and an intact EBA and to measure the gap between the DBBT stump and the radial tuberosity (Fig. 3). As described in MRI studies [13], we best viewed the EBA using ultrasound with the short-axis approach, and we could even directly assess whether or not it was intact using ultrasound and combining static and dynamic manoeuvres. In 30 of these 10 cases in which surgical procedure was delayed for over 15 days post-trauma, a significant thickening of the EBA was observed, most likely due to the increased biomechanical demand following the DBBT tear.

Another group of DBBT injuries relates to partial tears. Most partial tears, accounting for about 25% of total injuries (Table 1) encountered in our series occurred at the distal attachment of the DBBT to the radial tuberosity when just one or both DBBT components are partially detached. This partial tear type has been already described in MRI studies with a defined subgroup that includes low-grade or high-grade partial tears depending on whether they affect less or more than 50% of the total DBBT respectively (regardless of whether one or both components are affected) [14]. Ultrasound characterisation of these injuries is similar to MRI characterisation, which considers the following parameters: DBBT thinning, signal alteration at the attachment site, bicipitoradial bursitis and/or paratendinous effusion (Figs. 5 and 9) [40]. We were able to identify both of the abovementioned subgroups using ultrasound as the DBBT diameter can be easily measured in the short-axis view. We were only able to accurately identify and describe these cases by means of a meticulous examination in the long-axis and short-axis views and dynamic pronation–supination and flexion–extension manoeuvres. Specifically, dynamic manoeuvres were very important, as it was possible to observe the mobility of the intact portion and the lack of mobility of the torn portion. In our series, 16 out of 17 cases surgically verified as high-grade partial injuries had previously been correctly identified by ultrasound examination (Fig. 5), whereas only 7 out of 12 cases had previously been correctly identified by MRI examination. This indicates that ultrasound imaging could be more accurate than MRI in these cases.

However, another type of partial tear has been described in literature both by MRI and ultrasound, more accurately identified by ultrasound [10]. In these injuries, the tear affects only one of the two DBBT components (short head or long head) which becomes detached and retracted (Fig. 6). The anatomical basis of these partial injuries rests on the fact the DBBT is composed of two functionally and morphologically differentiated components, which have been detailed by both macroscopic and microscopic anatomy [9, 41, 42] and also demonstrated by ultrasound on normal volunteers [10]. In the present study, 3 of our patients were found to have a full-thickness tear and retraction of just one of the two DBBT components and therefore a partial tear, confirmed by posterior surgery (Figs. 6 and 7). The ultrasound examination of these injuries revealed similar findings to those encountered in complete tears, but in just one of the two DBBT components; a clear tendon rupture in both the long- and short-axis views and a refraction artefact at the tear point. Whether the tear affected the long or the short head, the unaffected component could be observed reaching and attaching to the radial tuberosity in the ultrasound examination.

Low-grade partial tears are the most difficult type of partial tears to be identified. They are characterised by hypoechogenic thickening of the 2–3 cm adjacent to the attachment site, with paratendinous effusion. This is accompanied by an alteration of the typical fibrillary echotexture and small focal hypoechogenic intrasubstance defects (Fig. 9). The main issue lies in the inability to establish whether these focal defects correspond to small intrasubstance tears or to the focus of mucoid degeneration by both ultrasound and MRI [43]. Problems associated with differentiating elongations (Figs. 7 and 8) from low-grade partial tear injuries (Fig. 9) were reflected in the fact that MRI did not confirm the ultrasound diagnosis in 27 out of 39 cases of minor injuries (in which surgery was not indicated). As we could not obtain surgical confirmation on which imaging method was more accurate, we could not fully elucidate this matter. In 7 out of 12 cases in which there was a discrepancy, ultrasound examination identified elongation injuries, whereas MRI identified low-grade partial tears. In 4 out of 12 cases, ultrasound examination identified low-grade partial tears, whereas MRI identified tendinopathy with no signs of tendon tear. In the remaining case of MRI/ultrasound disagreement, ultrasound identified a partial tear injury (type 2), whereas MRI identified a complete tear (type 3).

The present study has three main limitations. First, only patients with a clear alteration of the DBBT ultrasound pattern were considered for analysis and therefore we could not calculate the specificity of neither ultrasound nor MRI in detecting DBBT injuries. Second, as minor injuries were not clinically severe enough to justify surgical treatment, their ultrasound/MRI diagnosis could not be confirmed. Third, the population studied was not representative of the general population, as the patients consulting at our clinics correspond to people with company-paid health insurance; thus, their injuries are work-related.

Our results support the notion that ultrasound and MRI both have very good and similar sensitivities for identifying major DBBT injuries; complete tears with and without EBA tear and high-grade partial tears (types 3A, 3B, 2B and 2C). Although they agree in most cases, it remains uncertain whether ultrasound and/or MRI can more accurately differentiate low-grade partial tears at the attachment site (type 2A) from elongations (type 1A/B). Based on our findings, and those regarding previous MRI-based classification systems [14, 15], the ultrasound-based classification of DBBT traumatic injuries proposed herein could have implications for both diagnostic and therapeutic management, as it allows an accurate and early diagnosis to be made, and therefore it is at least as good as MRI (the gold standard at the moment) in terms of DBBT injury diagnosis. All and all, we recommend that ultrasound is considered as the first-line study in the evaluation of the DBBT injuries in those hospitals or clinics in which there is an ultrasonographer properly trained to perform such ultrasound examinations.
